# Curcumin Shows Antiviral Properties against Norovirus

**DOI:** 10.3390/molecules21101401

**Published:** 2016-10-20

**Authors:** Minji Yang, GilJae Lee, Jiyeon Si, Sung-Joon Lee, Hyun Ju You, GwangPyo Ko

**Affiliations:** 1 Department of Environmental Health Sciences, Graduate School of Public Health, Seoul National University, Seoul 151-742, Korea; yangmj@snu.ac.kr (M.Y.); meddugi1027@snu.ac.kr (G.L.); jane.jy.si@gmail.com (J.S.); 2 Department of Biotechnology, Graduate School of Life Sciences and Biotechnology, Department of Food Biosciences and Technology, College of Life Sciences and Biotechnology, Korea University, Seoul 151-742, Korea; junelee@korea.ac.kr; 3 N-Bio, Seoul National University, Seoul 151-742, Korea; gko@snu.ac.kr; 4 Institute of Health and Environment, Seoul National University, Seoul 151-742, Korea; happyrush@empas.com

**Keywords:** curcumin, norovirus, phytochemical

## Abstract

Phytochemicals provide environmentally friendly and relatively inexpensive natural products, which could potentially benefit public health by controlling human norovirus (HuNoV) infection. In this study, 18 different phytochemicals were evaluated for antiviral effects against norovirus using murine norovirus (MNV) as a model for norovirus biology. Among these phytochemicals, curcumin (CCM) was the most potent anti-noroviral phytochemical, followed by resveratrol (RVT). In a cell culture infection model, exposure to CCM or RVT for 3 days reduced infectivity of norovirus by 91% and 80%, respectively. To confirm the antiviral capability of CCM, we further evaluated its antiviral efficacy at various doses (0.25, 0.5, 0.75, 1, and 2 mg/mL) and durations (short-term: 10, 30, 60, and 120 min; long-term: 1, 3, 7, and 14 days). The anti-noroviral effect of CCM was verified to occur in a dose-dependent manner. Additionally, we evaluated the inhibitory effect of each phytochemical on the replication of HuNoV using a HuNoV replicon-bearing cell line (HG23). Neither CCM nor RVT had a strong inhibitory effect on HuNoV replication, which suggests that their antiviral mechanism may involve viral entry or other life cycle stages rather than the replication of viral RNA. Our results demonstrated that CCM may be a promising candidate for development as an anti-noroviral agent to prevent outbreaks of foodborne illness.

## 1. Introduction

Human norovirus (HuNoV) is the leading cause of viral gastroenteritis outbreaks worldwide [1,2,3]. Clinical symptoms of HuNoV infection include vomiting, nausea, and diarrhea within 24–48 h of infection [4]. HuNoV has low infectious doses of ~10 viral particles and is highly infectious to susceptible individuals. In particular, young children, the elderly, and people with weak immune systems experience severe symptoms [5,6]. HuNoV is transmitted mainly through the fecal-oral route and spreads easily from person to person, especially in crowded settings such as hospitals, restaurants, nursing homes, cruise ships, hotels, and schools [7,8,9].

Due to the current lack of a HuNoV vaccine, preventive measures are the most effective way to reduce outbreaks of HuNoV, which is known to be resistant to environmental stresses [10,11]. Therefore, current prevention methods for HuNoV include promoting personal hygiene for food handlers to avoid noroviral contamination and inactivation of infectious viral particles [12,13]. Inactivation of HuNoV relies on physical methods, such as heating or radiation, and chemical methods such as sodium hypochlorite or titanium dioxide [10,14,15,16]. However, the currently available methods have disadvantages for direct application to food. These include (1) increased cost of equipment and management; (2) potential acute and chronic toxic effects from chemical exposure; and (3) spoiling of food flavor or texture.

Therefore, environmentally friendly substances that might substitute for the established physical and chemical methods would be beneficial and directly applicable to food products such as oysters. Phytochemicals can be obtained from various natural plant extracts and most of them are “generally recognized as safe” (GRAS) [17]. This has led many researchers to study natural substances with antimicrobial properties [18,19]. Although previous studies have demonstrated that various phytochemicals possess antimicrobial effects, little research has been conducted to evaluate the efficacy of phytochemicals in reducing the infectiousness of norovirus [20].

Cultivation methods for HuNoV using B cells [21] or isolated enterocytes [22] were reported recently. Although those findings are very meaningful and can be a milestone to decipher the mechanisms of norovirus infection at a molecular level, it is a bit early to apply the methods to screening various antiviral compounds. The replication of HuNoV in a B-cell culture system did not appear to be robust, as observed in murine norovirus (MNV) [23], and the methods need to be validated with different types of HuNoV in other laboratories. Therefore, many previous studies have used viral models using MNV, feline calicivirus (FCV), and other in vitro models of HuNoV replication (e.g., HG23 cells) to evaluate the anti-noroviral activities of compounds [24,25]. In this study, we used MNV and HG23 cells to investigate the ability of several phytochemicals to inactivate norovirus.

We screened the anti-noroviral effects of 18 different phytochemicals using a viral model to study norovirus biology. Dose- and time-dependent trends were evaluated for the phytochemical with the greatest efficacy. Subsequently, the results of inactivation after long-term phytochemical incubation were fitted to three different mathematical models. Inhibitory effects on norovirus replication were measured using a HuNoV replicon-bearing cell (HG23) model.

## 2. Results

### 2.1. Evaluation of Phytochemical Cytotoxicity

Phytochemicals that had been previously reported to have antimicrobial effects were selected for analysis. Previously validated antiviral phytochemicals such as resveratrol (RVT) were included as positive controls [26]. The common names, abbreviations, and molecular weights of the phytochemicals tested in this study are summarized in Table A1. The cells were treated with phytochemicals and incubated for 1 h (RAW 264.7 cells) and 72 h (HG23 cells) at 37 °C with 5% CO_2_. DMSO was used as a vehicle control. The viability of cells (RAW 264.7 and HG23 cells) after treatment with phytochemicals was evaluated using the water-soluble tetrazolium salt (WST) assay and was compared to a vehicle control. In RAW 264.7 cells, phytochemicals showed no cytotoxicity at a concentration of 0.1 mg/mL (Table 1). On the other hand, the viability of HG23 cells fell to below 80% after treatment with most phytochemicals at this concentration (data not shown). Because of the susceptibility of this cell line, HG23 cells were exposed to various concentrations of phytochemicals to find the maximum concentration with no significant cytotoxicity. We found that for most phytochemicals, a concentration of 20 μg/mL was optimal to assess antiviral activity without significant cytotoxicity in HG23 cells. Among the 18 tested phytochemicals, ellagic acid (ELG), curcumin (CCM), RVT, and compound K (CK) showed cytotoxicity at 20 μg/mL, so these four compounds were tested at 2 μg/mL in the assay of anti-noroviral activity.

### 2.2. Effects of Phytochemicals on Neutralization of MNV

**Figure 1:**
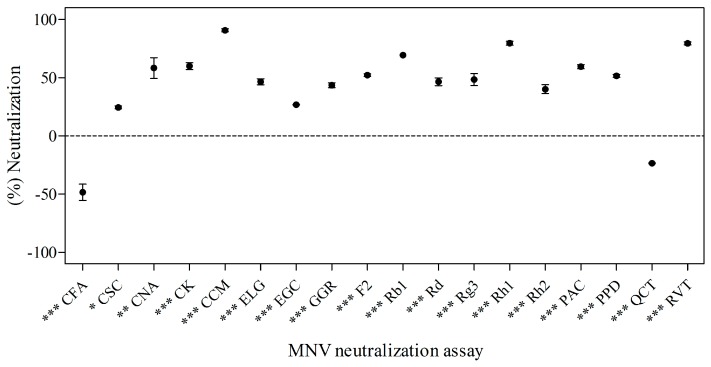
The effect of phytochemicals on murine norovirus (MNV) neutralization in RAW 264.7 cells. MNV was mixed with 1 mg/mL of 18 phytochemicals and stored for 72 h at 4 °C. DMSO was used as a vehicle control and virus titer was measured by plaque assay. The mean diameter of plaques was 1 mm, and visible plaques were counted 72 h after infection. Each point was derived from triplicate determinations. Results are expressed as percentage neutralized virus compared to a vehicle control (mean ± SEM). Statistical significance was determined based on ANOVA with Bonferroni post-tests. * *p* < 0.05; ** *p* < 0.01; *** *p* < 0.001 compared to vehicle control.

MNV was treated with selected phytochemicals (a final concentration of 1.0 × 10^6^ plaque-forming units (PFU)/mL and 1 mg/mL of phytochemical) at 4 °C for 3 days. Quantification of neutralized virus was measured by plaque assay. The anti-noroviral activity of the 18 tested phytochemicals is described in Figure 1. Phytochemicals were generally effective on neutralizing MNV, with the exception of quercetin (QCT) and caffeic acid (CFA). Among them, CCM was the most effective anti-noroviral phytochemical (Figure 1). Additionally, capsaicin (CSC) (24.14% ± 2.28%; *p* < 0.05) and cinnamic acid (CNA) (60.19% ± 22.38%; *p* < 0.01) significantly inhibited the virus. Protopanaxadiol (PPD) (52.14% ± 2.28%), CK (60.19% ± 6.67%), ginsenoside Rh2 (Rh2) (39.74% ± 6.67%), ginsenoside Rg3 (Rg3) (48.71% ± 8.80%), ginsenoside F2 (F2) (52.14% ± 2.28%), ginsenoside Rd (Rd) (46.30% ± 6.67%), ginsenoside Rb1 (Rb1) (69.10% ± 0.00%), ginsenoside Rh1 (Rh1) (80.05% ± 6.67%), 10-gingerol (GGR) (43.77% ± 4.50%), ELG (46.30% ± 6.67%), proanthocyanidin (PAC) (59.26% ± 4.50%), CCM (90.88% ± 10.87%), RVT (79.58% ± 6.67%), and epigallocatechin gallate (EGC) (27.56% ± 0.00%) (*p* < 0.001) also significantly neutralized MNV. On the other hand, CFA and QCT did not show antiviral activity against MNV. CCM and RVT showed 90.88% ± 10.87% and 79.58% ± 6.67% neutralization of virus, which corresponded to 1.04 log PFU/mL reduction and RVT 0.69 log PFU/mL reduction, respectively.

### 2.3. Dose-Response Relationship between CCM Exposure and Inactivation of MNV

**Figure 2:**
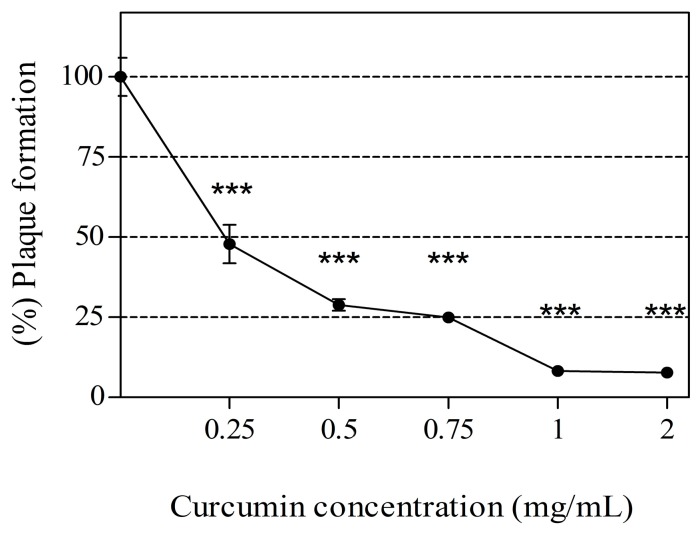
Anti-noroviral effect of curcumin (CCM) at various concentrations in RAW 264.7 cells. MNV was mixed with 0.25, 0.5, 0.75, 1 or 2 mg/mL CCM and stored 72 h at 4 °C. Virus titers were measured by plaque assay and percentage was compared to a vehicle control (DMSO treated). Statistical significance was determined based on ANOVA with Bonferroni post-tests. Each point was derived from triplicate determinations (mean ± SEM). *** *p* < 0.001 comparing to vehicle control.

The anti-noroviral activity of CCM across various concentrations (0.25, 0.5, 0.75, 1, and 2 mg/mL) is shown in Figure 2. Cytotoxicity was not observed at those concentrations (data not shown). The anti-noroviral activity of CCM was dose dependent. CCM at 0.25, 0.5, 0.75, 1, and 2 mg/mL neutralized MNV by 52.85% ± 11.39%, 68.35% ± 5.42%, 68.96% ± 3.13%, 89.99% ± 5.88%, and 90.43% ± 9.13%, respectively.

### 2.4. Short- and Long-Term Effects of Curcumin on Viral Neutralization

**Figure 3:**
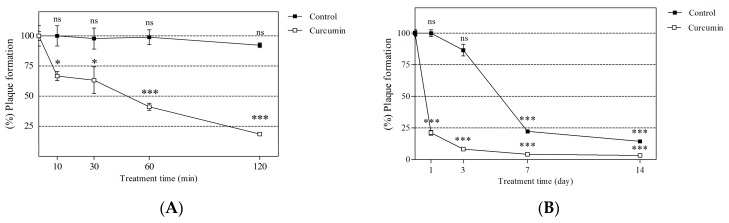
Anti-noroviral efficacy of CCM under short- and long-term incubation in RAW 264.7 cells. MNV was mixed with 1 mg/mL of CCM and stored for (**A**) short (10, 30, 60, and 120 min) and (**B**) long periods (1, 3, 7 and 14 days) at 4 °C. Virus titers were measured by plaque assay (DMSO as a vehicle control). Percentage of plaque formation was compared to the initial time (day 0). Statistical significance was determined based on ANOVA with Bonferroni post-tests. Each point represents the mean ± SEM of triplicate determination. * *p* < 0.05; *** *p* < 0.001 comparing to vehicle control.

The anti-noroviral activity of CCM was determined after various incubation durations (Figure 3), and showed a time-dependent response to short-term incubation (Figure 3A). The anti-noroviral effects of CCM occurred time-dependent manner. At 10, 30, 60, and 120 min of incubation, MNV was reduced by 33.33% ± 3.85%, 36.79% ± 11.13%, 58.89% ± 2.94%, and 82.50% ± 1.21%, respectively. After 1, 3, 7 and 14 days of incubation, MNV was reduced by 78.87% ± 1.86%, 92.87% ± 1.03%, 95.88% ± 0.52%, and 96.78% ± 0.46%, respectively (Figure 3B). In case of long-term incubation, neutralization of virus over time was observed in a DMSO-treated vehicle control at day 7 and 14. Even though the results indicated that virus particles decayed naturally at these time points, CCM significantly neutralized virus particles compared to a vehicle control at day 7 (*p* < 0.001) and 14 (*p* < 0.01).

### 2.5. Model Evaluation with Experimental Data

**Figure 4:**
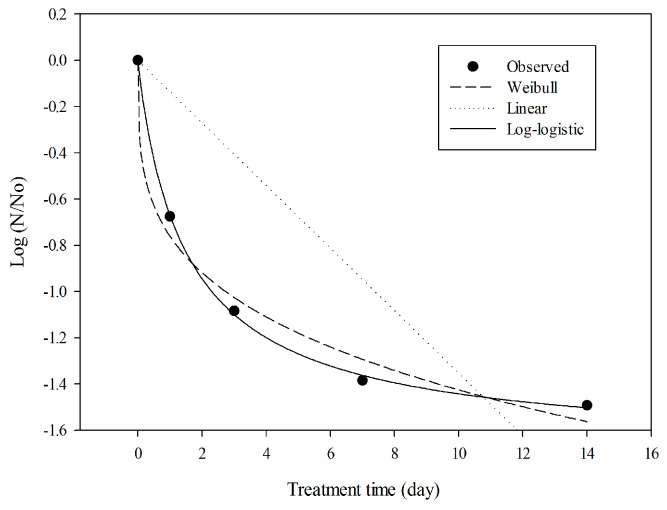
Data fitting of the inactivation curve for MNV under long-term incubation. MNV was mixed with 1 mg/mL of CCM and stored for 1, 3, 7 or 14 days at 4 °C. Data were fitted to several different models.

To assess the anti-noroviral behavior of CCM over a long period (days) (Figure 3B), three different models were evaluated: (i) the linear model; (ii) the Weibull model; and (iii) the log-logistic model (Figure 4). To assess the fit of the models, regression coefficient (R^2^), root-mean-square error (RMSE), and Akaike information criterion (AIC) were compared, and are summarized in Table 2. Higher R^2^ and lower RMSE values indicate a better-fitting model [27]. The log-logistic model showed the best fit to CCM data, with the highest R^2^ (0.99) and lowest RMSE (0.02) values. In contrast, the linear model appeared to be inappropriate for the observed data.

### 2.6. Effects of Phytochemicals on Replication of HuNoV in Replicon-Bearing Cells

**Figure 5:**
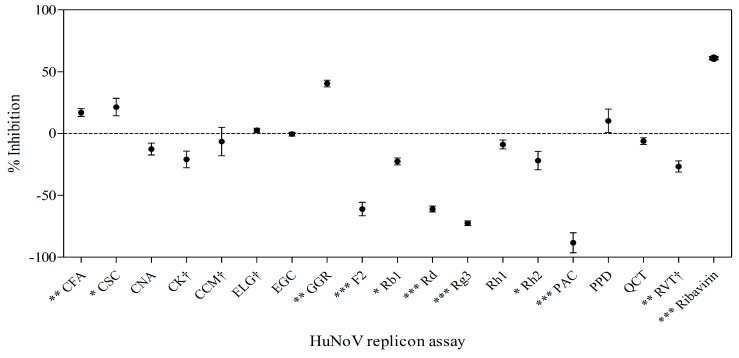
The effect of phytochemicals on human norovirus (HuNoV) replication using a replicon-bearing cell line (HG23). HG23 cells were treated with 20 µg/mL of phytochemicals and incubated for 72 h at 37 °C. ^†^ Treatment concentration is 2 µg/mL due to cytotoxicity at 20 µg/mL. Replicons were measured using real-time RT-PCR and normalized with β-actin. DMSO and ribavirin (200 μM) were used as a vehicle and positive control, respectively. Each point consists of values derived from triplicate determinations. Results are expressed as percentage inhibition (mean ± SEM). Statistical significance was determined based on ANOVA with Bonferroni post-tests. * *p* < 0.05; ** *p* < 0.01; *** *p* < 0.001 comparing to vehicle control.

To assess whether phytochemicals inhibit HuNoV replication, we performed an anti-noroviral screening assay using the HG23 cell line [25]. Cells were treated with phytochemicals, and replicon RNA levels were assessed 72 h later by real-time RT-PCR. The results are shown in Figure 5. GGR (40.50% ± 3.83%; *p* < 0.01), CFA (17.07% ± 3.95%), and CSC (35.60% ± 8.50%; *p* < 0.05) significantly inhibited replicon RNA levels (Figure 5). CCM ((+) 6.47% ± 9.33%) and RVT ((+) 26.62% ± 4.70%) did not show inhibitory effects on replication. Furthermore, 7 of 18 (Rh2, Rg3, F2, Rd, Rb1, PAC, and RVT) phytochemicals increased replicon RNA levels. Ribavirin (200 μM), which was used as a positive control, reduced replicon levels by 33%.

## 3. Discussion

Studies on antimicrobial agents derived from phytochemicals aim to find natural products for preventing viral infection. For thousands of years, humans have consumed a variety of plant extracts as traditional medicine, natural therapies, and phytochemicals. The advantages of plant extracts in the prevention and treatment of infectious diseases include cost-effectiveness and safety, as compared to synthetic chemical antimicrobials and disinfectants. However, studies on the effectiveness of plant extracts against norovirus are limited and focus on specific phytochemical compounds such as tannins and flavonoids from berries and other fruits [28,29].

In the present study, we investigated the anti-noroviral effect of 18 phytochemicals in terms of neutralizing viral particles and inhibiting viral replication in two models: MNV and HuNoV replicon-bearing cells (HG23). These models were used due to the difficulty of HuNoV culture. Ten different food-derived phytochemicals and eight ginsenosides with different glycosidic moieties were selected based on previous reports of potent antibacterial and/or antiviral activities. Given that the efficacy of phytochemical compounds is greatly affected by slight modification of their structure (e.g., glycosylation, methylation, acetylation, etc.), we analyzed several ginsenosides to determine whether the antiviral effects were influenced by the modified glycosyl ligands.

In the MNV neutralization assay, the most effective phytochemical was found to be CCM, which reduced the titer of MNV by 90.88% ± 10.87%. CCM is derived from the spice turmeric, and has clinical importance in improving cardiovascular and neurological health, as well as fighting breast tumors and gastric inflammation [30,31,32]. Dietary intake of curcumin was reported up to 200 mg/day in some Asian countries where curcumin has been widely used as a common constituent in food (e.g., curry powder) and traditional medicine [33]. In a recent clinical study, participants taking 4 g of mixed curcuminoids (2920 mg curcumin) daily for 6 days showed 363 ng/mL of curcumin in rectal tissue at day 7 [34].

Several studies have reported antiviral effects of CCM. For example, it affected hepatitis C virus (HCV) envelope fluidity without changing virus integrity, resulting in inhibition of binding and fusion to the host [35]. Other studies showed that CCM inhibited HCV using a replicon model [36] and also reduced herpes simplex virus (HSV) [32,37]. As far as we know, two groups have reported anti-noroviral effects of CCM recently [38,39]. Photodynamically activated CCM was used in both reports and significantly neutralized MNV with damage to nucleic acid. The neutralized assay against virus was performed at room temperature or 37 °C. Considering norovirus is more stable at low temperature [40], and most outbreaks were prevalent in winter [41], we targeted 4 °C for the MNV neutralization assay.

CCM was investigated at various concentrations and anti-noroviral effects occurred in a dose-dependent manner. In our study, MNV particles were first treated with the phytochemicals, and the mixture with neutralized virus and phytochemicals was infected to host cells. There may be a possibility that the phytochemical itself affects the infection of host cells, not the neutralized viral particles. However, CCM showed a time-dependent inactivation of MNV and this suggests that the effect was based on increased neutralized virus by incubation with CCM rather than direct influence of CCM on host cells. 

Short-term incubation with CCM (10, 20, 60 and 120 min) led to gradual reduction of MNV, with an 82.50% ± 1.21% reduction after 120 min. In the long-term test (1, 3, 7 and 14 days), a sharp decrease in MNV was observed within 1 day (78.87% ± 1.86% reduction). Comparing the results of 120 min and 1 day incubation, it is apparent that the major decrease occurred within the first 2 h. According to the *D*-value (time in days required to inactivate 90% of the MNV) based on long-term data, 2.30 days of incubation are required to inactivate 90% of MNV. In control treatments, MNV reduction was about 80% after 7 days. The same trend has been observed in other studies. MNV titers decreased by 0.96 log after 7 days at 4 °C [42], likely due to natural particle decay. RVT, a polyphenol abundant in grape seeds and skin, is capable of neutralizing MNV when incubated with the virus [26]. Our results demonstrated that RVT significantly neutralized MNV (79.58% ± 6.67%) but had no inhibitory effect in the replicon model ((+) 26.62% ± 4.70%). PAC and CNA also significantly reduced MNV by approximately 60%, in agreement with previous studies showing that PAC decreased the levels of HuNoV surrogates such as FCV and MNV [18,43,44]. In the case of CNA, this is the first report of an anti-noroviral effect. Su et al. studied the anti-noroviral effects of L-epicatechin using MNV and feline calicivirus (FCV-F9) [11]. The exposure of 0.5 mM l-epicatechin for 2 h at 37 °C neutralized only FCV-F9 by 1.40 log PFU/mL. In this study, EGC, a type of catechin, significantly inactivated MNV (26.83% ± 0.33%). This might be due to the differences of a chemical structure of catechin or exposure conditions.

In this study, CSC and GGR exhibited inhibitory effects in both models. CSC is a constituent of red peppers. CSC neutralized MNV (24.14% ± 2.28%) and reduced replicon RNA level (35.60% ± 8.50%). CSC has previously shown anti-microbial activity against bacterial pathogens including *Helicobacter pylori* [45,46] and *Staphylococcus aureus* [47]. CSC also reduced the mortality of HSV-infected mice [48] and has been hypothesized to inhibit virus–neuron interaction [49]. This study is the first report of an anti-noroviral effect of CSC. GGR is a culinary spice and is a common medicinal plant in China. GGR is an active constituent of most ginger species, and has been reported to have antimicrobial effects against *Helicobacter pylori* and periodontal bacteria [50,51]. In this study, we found that GGR had anti-noroviral activity in both models. GGR neutralized MNV (43.77% ± 4.50%) and inhibited the replication of norovirus (40.50% ± 3.83%) in the HG23 replicon model. This implies that GGR is a potential anti-noroviral for both in vitro inactivation of norovirus particles (high dose) and inhibition of norovirus replication in cells (low dose).

Ginsenosides are active constituents of most ginseng species. In the MNV neutralization assay, most ginsenosides showed an antiviral effect. Rh1 (80.05% ± 6.67%) and Rb1 (69.10% ± 0.00%) were particularly effective. However, five ginsenosides (F2, Rh2, Rd, Rg3, Rb1) increased, rather than repressed, the replication of norovirus in the HG23 replicon model. This result may be explained by the role of ginsenosides in the cholesterol biosynthesis pathway. It has been reported that the inhibition of cholesterol synthesis using statins (HMG-CoA reductase inhibitors) significantly increased HuNoV replicon RNA and proteins in the HG23 replicon model [52]. Ginsenosides also suppress cholesterogenesis and lipogenesis [53]. HMG-CoA reductase activity was significantly downregulated after treatment with ginseng powder. Our data are consistent with previous findings, in that the inhibition of cholesterol biosynthesis by ginsenosides significantly increased the level of norovirus RNA in the HG23 replicon model. Thus, it is important to consider the various stages of viral infection when evaluating the anti-noroviral effects of phytochemicals.

In summary, we evaluated 18 different types of phytochemicals and identified CCM as having the strongest anti-noroviral effects. This study indicated that phytochemicals such as CCM hold promise as natural anti-noroviral agents for the food-processing industry.

## 4. Materials and Methods

### 4.1. Tested Phytochemicals

A total of 18 phytochemicals were purchased from various research institutes and manufacturers. The ginsenosides PPD, CK, Rg3, and Rb1 were purchased from the Ambo Institute (Daejeon, Korea) and ginsenoside Rd was provided by BTGin (Daejeon, Korea). Ginsenosides Rh2, F2, and Rh1, and ellagic acid (ELG) were obtained from Chengdu Biopurify Phytochemicals Ltd. (Chengdu, Sichuan, China). Gingerol (GGR) and proanthocyanidin (PAC) were provided by Wuhan ChemFaces Biochemical Co., Ltd. (Wuhan, Hubei, China). Curcumin (CCM), resveratrol (RVT), cinnamic acid (CNA), caffeic acid (CFA), epigallocatechin gallate (EGC), capsaicin (CSC), and quercetin (QCT) were obtained from Sigma Aldrich (St. Louis, MO, USA). Phytochemicals were dissolved in dimethyl sulfoxide (DMSO) (Sigma Aldrich) and stored at 4 °C until use.

### 4.2. Virus and Cell Lines

MNV was obtained from Dr. Virgin’s laboratory (School of Medicine, Washington University, St. Louis, MO, USA) and was cultivated following previously published protocols [10]. HG23 cells were kindly provided by Dr. Chang’s laboratory (College of Veterinary Medicine, Kansas State University, Manhattan, KS, USA). Both RAW 264.7 and HG23 cells were maintained in Dulbecco’s modified Eagle’s medium (DMEM) (Gibco, Grand Island, NY, USA) containing 10% fetal bovine serum (Gibco), 10 mM nonessential amino acids (Gibco), 10 mM sodium bicarbonate (Gibco), 10 mM HEPES (Gibco), and 50 µg/µL gentamicin (Gibco). Cells were cultured in 5% CO_2_ at 37 °C in an incubator. The antibiotic G418 (0.5 mg/mL) was added to the media for HG23 cell culture only.

### 4.3. Cultivation of MNV and Preparation of Viral Stock

MNV was inoculated onto confluent RAW 264.7 cells and incubated until the virus caused visible cytopathic effects [14,54]. Infected cells were subjected to three freeze–thaw cycles. Propagated MNV was purified with chloroform (AMRESCO, Solon, OH, USA) and centrifuged at 5000× *g* for 20 min at 4 °C. Subsequently, the supernatant was collected and centrifuged through a 0.2-micron filter (Amicon Ultra-15; Millipore, Billerica, MA, USA). After concentration, the supernatant was aliquoted and stored at −80 °C.

### 4.4. Cytotoxicity Assay

To measure cytotoxicity, we performed the water-soluble tetrazolium salt (WST) assay using an EZ-CyTox kit (Daeil Lab, Seoul, Korea) [55]. Concentrations of phytochemicals were adjusted to 0.1 mg/mL. Diluted phytochemicals were added to RAW 264.7 cells, with a maximum concentration of 0.1 mg/mL. For HG23 cells, 20 µg/mL and 2 µg/mL of phytochemicals were used for cytotoxicity, respectively. RAW 264.7 and HG23 cells were seeded onto 96-well plates and incubated at 37 °C in a 5% CO_2_ incubator overnight. The media were carefully aspirated, then the cells were treated with phytochemicals and incubated for 1 h (RAW 264.7) and 72 h (HG23) at 37 °C with 5% CO_2_. DMSO was used as a control. The cells were then treated with EZ-Cytox solution and incubated again for 2 h. After incubation, phytochemical-treated cells were compared with DMSO-treated cells (vehicle control). Cytotoxicity was measured using a microplate reader (TECAN, Männedorf, Switzerland) at 450 nm.

### 4.5. Quantification of MNV by Plaque Assay

The titer of MNV (PFU/mL) was quantified by a plaque assay [56]. RAW 264.7 cells were seeded onto 6-well plates at 3 × 10^6^ cells/well and incubated at 37 °C in a 5% CO_2_ incubator overnight. Ten-fold serial dilutions of MNV were prepared in DMEM. RAW 264.7 cells were inoculated with 500 µL of MNV solution. The plates were incubated for 1 h and gently rocked every 15 min. After incubation, the inocula were aspirated and the cells were subsequently overlaid with 3 mL of a 1:1 (*v*/*v*) mixture of 1.5% SeaPlaque agarose (Lonza, Rockland, ME, USA) and 2× minimum essential medium (MEM) supplemented with 10 mM nonessential amino acids (Gibco), 10 mM HEPES (Gibco), 10 mM sodium bicarbonate (Gibco), 50 µg/µL gentamycin (Gibco), and 10% fetal bovine serum (Gibco) at 37 °C. Plates were incubated for another 72 h, then plaques were counted and recorded in PFU/mL.

### 4.6. MNV Neutraization Asay

MNV stock (50 µL) was treated with 2.5 µL of the selected phytochemical, followed by addition of 447.5 µL of DMEM (a final concentration of 1.0 × 10^6^ PFU/mL and 1 mg/mL of phytochemical) at 4 °C for 3 days. This neutralization study was based on a modified procedure described in a previous study [17]. MNV is more stable at 4 °C than at room temperature (25 °C). Thus, we exposed MNV to phytochemicals at 4 °C, following the previously published protocol [10]. The efficacy of neutralization against MNV was quantitatively evaluated for each of the 18 phytochemicals tested over several incubation periods. For short-term exposure tests, we applied the phytochemicals to MNV for 10, 30, 60, or 120 min. Additionally, we performed long-term exposure assays by applying the phytochemicals tested to MNV for 1, 3, 7, and 14 days [42]. Plaque assays were performed as described above. Each experiment was tested in triplicate.

### 4.7. HuNoV Replication Assay Using HuNoV Replicon-Bearing HG23 Cells

The effect of phytochemicals on virus replication was quantitatively evaluated using HuNoV (Norwalk) replicon-bearing HG23 cells and a real-time RT-PCR assay [57]. HG23 cells (2 × 10^5^ cells/well) were seeded into six-well plates in complete DMEM with G418, then cultivated at 37 °C in a 5% CO_2_ incubator for 24 h. Subsequently, phytochemicals were added to the HG23 cells, which were incubated for a further 72 h. The supernatant was removed, and the remaining cells were collected for RNA-load quantification by real-time RT-PCR. To normalize intracellular viral RNA load, β-actin mRNA was used, and the viral replicon/β-actin ratio was calculated using the Pfaffl method [58], as follows: E_Norwalk_^ΔCT,Norwalk(control−sample)^/E_β-actin_^ΔCT,β-actin(control−sample)^, where E refers to the real-time RT-PCR amplification efficiency, E = 10(−1/slope). ΔC_T_ is the difference in C_T_ (threshold cycle) values.

### 4.8. RNA Extraction and Real-time RT-PCR

To extract total RNA from HG23 cells, an Easy-spin Total RNA extraction kit (iNtRON, Seoul, Korea) was used according to the manufacturer’s protocol. For detection of Norwalk virus replicon RNA, forward (5′-GCY ATG TTC CGY TGG ATG-3′) and reverse (5′-GTC CTT AGA CGC CAT CAT CAT-3′) primers and a probe (5′-VIC-TGT GGA CAG GAG ATC GC-MGB-3′) were used as described previously [59]. Similarly, for detection of β-actin mRNA, previously described forward (5′-GGC ATC CAC GAA ACT ACC TT-3′) and reverse (5′-AGC ACT GTG TTG GCG TAC AG-3’) primers and a probe (5′-5-FAM-ATC ATG AAG TGT GAC GTG GAC ATC CG-BHQ1-3′) were used [60]. Duplex real-time RT-PCR assays were carried out using an AgPath-ID One Step RT-PCR kit (Ambion, Austin, TX, USA) with a 25 μL reaction mixture containing 12.5 μL of 2× RT-PCR Buffer, 1 μL of 25× RT-PCR Enzyme Mix, 2.5 μL of template RNA, 200 nM Norwalk virus replicon primers and 100 nM probe, and 200 nM β-actin primers and 100 nM probe. Cycling conditions were reverse transcription at 42 °C for 30 min, initial denaturation at 95 °C for 10 min, and 45 cycles of denaturation at 95 °C for 15 s, annealing, and extension at 60 °C for 1 min (ABI 7300 Real-Time PCR System; Applied Biosystems, Foster City, CA, USA). For absolute quantitation of the Norwalk virus replicon, 10-fold dilutions of a known concentration of the norovirus GI type or the β-actin clone were used to construct standard curves.

### 4.9. Fitting the Experimental Data to Three Different Models

To predict the anti-noroviral effect of CCM and understand the inactivation kinetics based on a long exposure time (days), three models (linear, Weibull, and log-logistic) were compared to determine which best fitted the data. Neutralization curves were fitted to the observed data in SigmaPlot (ver. 2.0; Systat Software, San Jose, CA, USA). To evaluate the models, the regression coefficient (R^2^), root-mean-square error (RMSE), and Akaike information criterion (AIC) of each model were compared. The linear model is a general model for explaining the inactivation of microorganisms under constant conditions [27], and is calculated as follows:

(1) $$log\frac{N_{t}}{N_{0}}=-\frac{t}{D}$$

where *N_t_* is the concentration of MNV (PFU/mL) after exposure, *N*_0_ is the initial concentration of MNV (PFU/mL), *t* signifies exposure time, and D denotes the *D*-value, or the time in days required to inactivate 90% of the MNV under the indicated conditions. The Weibull model is a nonlinear model with a sigmoidal shape [61], calculated as follows:

(2) $$log\frac{N_{t}}{N_{0}}=-bt^{n}$$

where *t* indicates the exposure time, and *b* is a scaling parameter. The parameter *n* alters the shape of the curve: if *n* > 1, it is downward concave (shoulder) and if *n* < 1, it is upward concave (tailing). The log-logistic model was first proposed by Cole et al. to describe nonlinear inactivation of microorganisms [62], and has been modified to decrease the number of parameters [63]. The log-logistic model is defined as follows:

(3) $$log\frac{N_{t}}{N_{0}}=\frac{A}{1+e^{4\sigma \left(\tau -logt\right)/A}}-\frac{A}{1+e^{4\sigma \left(\tau -logt_{0}\right)/A}}$$

where *A* is the difference between the upper and lower asymptotes (log PFU/mL), σ is the maximum inactivation rate (log (PFU/mL)/log day), and τ denotes time to the maximum inactivation rate (log day). Since log (*t*_0_) at *t* = 0 is not defined, a small value of *t*_0_ (*t* = 10^−6^ day) was used instead of 0.

### 4.10. Statistical Analysis

The data were transformed to a percentage scale to quantify viral neutralization, expressed as inhibition (%). Statistical significance was determined based on one-way or two-way analysis of variance (ANOVA) with Bonferroni post-tests, using Prism 5 software (Graph-Pad Software, San Diego, CA, USA).

## Figures and Tables

**Table 1:** Evaluation of cytotoxicity of phytochemicals in two different cell lines.

Common Name	Abbreviation	RAW 264.7 ^a^	HG23 ^b^
		% Cell Viability	
Caffeic acid	CFA	101.14 ± 6.00	92.62 ± 3.87
Capsaicin	CSC	119.18 ± 2.83	82.92 ± 0.58
Cinnamic acid	CNA	102.25 ± 4.11	93.87 ± 6.75
Compound K	CK	91.89 ± 1.10	80.67 ± 3.07 ^†^
Curcumin	CCM	102.14 ± 1.89	92.57 ± 2.41 ^†^
Ellagic acid	ELG	80.32 ± 1.68	89.91 ± 7.50 ^†^
Epigallocatechin gallate	EGC	94.44 ± 5.55	87.44 ± 3.09
10-Gingerol	GGR	83.68 ± 3.03	86.07 ± 7.02
Ginsenoside F2	F2	88.30 ± 3.65	101.52 ± 4.22
Ginsenoside Rb1	Rb1	95.27 ± 5.31	101.48 ± 4.61
Ginsenoside Rd	Rd	87.17 ± 0.79	102.11 ± 2.80
Ginsenoside Rg3	Rg3	84.75 ± 2.10	95.29 ± 3.47
Ginsenoside Rh1	Rh1	98.54 ± 2.94	94.38 ± 4.51
Ginsenoside Rh2	Rh2	95.32 ± 2.00	94.18 ± 4.86
Proanthocyanidin	PAC	87.47 ± 7.31	102.40 ± 0.57
Protopanaxadiol	PPD	97.57 ± 1.80	99.03 ± 3.17
Quercetin	QCT	127.26 ± 3.65	88.50 ± 7.23
Resveratrol	RVT	84.46 ± 2.03	80.07 ± 5.94 ^†^

^a^ RAW 264.7 cells were treated with 100 µg/mL of phytochemical and incubated for 1 h and ^b^ HG23 cells were treated with 20 µg/mL and incubated for 72 h at 37 °C with 5% CO_2_. ^†^ Treatment concentration is 2 µg/mL due to cytotoxicity at 20 µg/mL. DMSO was used as a vehicle control. All data (mean ± SEM) were derived from triplicate determination.

**Table 2:** Comparison of linear, Weibull, and log-logistic models in terms of long-term anti-noroviral data for curcumin.

Fitting Model	Curcumin Inactivation			
	R^2^	RMSE	AIC	*D*-value
Linear distribution	0.25	0.53	2.40	7.39
Weibull distribution	0.98	0.09	−10.94	2.72
Log-logistic distribution	0.99	0.02	−22.05	2.30

R^2^ is the correlation coefficient, RMSE is the root mean square error, AIC is the Akaike information criterion, and *D*-value is the time in days required to inactivate 90% of the MNV.

## Appendix

**Table A1:** List of phytochemicals tested for anti-noroviral activity.

Name of Phytochemical	Abbreviation	Molecular Weight	Sources
Protopanaxadiol	PPD	460.73	ginseng
Compound K	CK	653.81	ginseng
Ginsenoside Rh2	Rh2	622.87	ginseng
Ginsenoside Rg3	Rg3	785.01	ginseng
Ginsenoside F2	F2	785.01	ginseng
Ginsenoside Rd	Rd	947.15	ginseng
Ginsenoside Rb1	Rb1	1109.30	ginseng
Ginsenoside Rh1	Rh1	638.87	ginseng
Curcumin	CCM	368.38	turmeric
Resveratrol	RVT	228.24	grape and berries
Cinnamic acid	CNA	148.16	cinnamon
Caffeic acid	CFA	180.16	coffee
Epigallocatechin gallate	EGC	458.37	green tea
Capsaicin	CSC	305.41	red pepper
Quercetin	QCT	302.24	onions and apples
10-Gingerol	GGR	350.50	ginger
Proanthocyanidin	PAC	578.52	fruits and berries
Ellagic acid	ELG	302.19	fruits and berries
